# Comprehensively prognostic and immunological analysis of snail family transcriptional repressor 2 in pan-cancer and identification in pancreatic carcinoma

**DOI:** 10.3389/fimmu.2023.1117585

**Published:** 2023-05-11

**Authors:** Dandan Zhang, Zhenhong Jiang, Jianping Hu, Xiaoyun Sun, Yan Zheng, Yang Shen

**Affiliations:** ^1^ Department of General Surgery, the Second Affiliated Hospital of Nanchang University, Nanchang, Jiangxi, China; ^2^ Jiangxi Key Laboratory of Molecular Medicine, the Second Affiliated Hospital of Nanchang University, Nanchang, China; ^3^ Department of Medical Genetics, the Second Affiliated Hospital of Nanchang University, Nanchang, China

**Keywords:** snail family transcriptional repressor 2 (SNAI2), tumor immunity, pan-cancer, prognosis biomarker, pancreatic carcinoma

## Abstract

**Background:**

Snail family transcriptional repressor 2 (SNAI2) is a transcription factor that induces epithelial to mesenchymal transition in neoplastic epithelial cells. It is closely related to the progression of various malignancies. However, the significance of SNAI2 in human pan-cancer is still largely unknown.

**Methods:**

The Cancer Genome Atlas (TCGA), Genotype-Tissue Expression (GTEx), and Cancer Cell Line Encyclopedia (CCLE) databases were taken to examine the SNAI2 expression pattern in tissues and cancer cells. The link between SNAI2 gene expression levels and prognosis, as well as immune cell infiltration, was investigated using the Kaplan-Meier technique and Spearman correlation analysis. We also explored the expression and distribution of SNAI2 in various tumor tissues and cells by the THPA (Human Protein Atlas) database. We further investigated the relationship between SNAI2 expression levels and immunotherapy response in various clinical immunotherapy cohorts. Finally, the immunoblot was used to quantify the SNAI2 expression levels, and the proliferative and invasive ability of pancreatic cancer cells was determined by colony formation and transwell assays.

**Results:**

We discovered heterogeneity in SNAI2 expression in different tumor tissues and cancer cell lines by exploring public datasets. The genomic alteration of SNAI2 existed in most cancers. Also, SNAI2 exhibits prognosis predictive ability in various cancers. SNAI2 was significantly correlated with immune-activated hallmarks, cancer immune cell infiltrations, and immunoregulators. It’s worth noting that SNAI2 expression is significantly related to the effectiveness of clinical immunotherapy. SNAI2 expression was also found to have a high correlation with the DNA mismatch repair (MMR) genes and DNA methylation in many cancers. Finally, the knockdown of SNAI2 significantly weakened the proliferative and invasive ability of pancreatic cancer cells.

**Conclusion:**

These findings suggested that SNAI2 could be used as a biomarker in human pan-cancer to detect immune infiltration and poor prognosis, which provides a new idea for cancer treatment.

## Introduction

Malignancies have increased at an alarming rate over the last few decades, becoming one of the major causes of death worldwide, while also significantly increasing the public health burden (1). Blocking immunological checkpoints such as cytotoxic T lymphocyte-associated protein 4 (CTLA-4), programmed cell death ligand 1 (PD-L1), and programmed cell death protein-1 (PD-1) (2, 3), has made a great contribution to the immunotherapy of various cancers in recent years. However, despite the success of ICIs, resistance to these agents restricts the number of patients able to achieve durable responses, and immune-related adverse events complicate treatment. Thus, a better understanding of the requirements for an effective and safe antitumor immune response following ICI therapy is needed (4). Despite considerable efforts by scientists to enhance the diagnosis and treatment of cancer, the treatment of cancer still imposes a huge economic burden on countries around the world (5). Therefore, it is particularly important to explore new immunotherapy biomarkers or new immunoregulatory genes for cancer patients.

Snail family transcriptional repressor 2 (SNAI2) (6), also known as SLUG, is a member of the Snail/Scratch superfamily, which also includes SNAI1, SNAI3, and SCRTs, among others. SNAI2 is currently receiving a lot of interest as a promising biomarker and a key mediator in a variety of human malignancies. As an epithelial-to-mesenchymal transition transcriptional factor, SNAI2 enhances cell polarity and adhesion loss while providing migratory and invasive characteristics (7). SNAI2 cloud promotes the invasion of ovarian cancer cells by upregulating MARCKS expression (8) and through regulating ferroptosis (9). A similar study suggested that ASB13 inhibits breast cancer metastasis by promoting SNAI2 degradation (10). In addition, SNAI2 upregulation is associated with an aggressive phenotype in fulvestrant-resistant breast cancer cells (11). And in glioma stem cells, SNAI2 exerts pro-tumorigenic effects *via* the PHLPP2-mediated Akt pathway (12). In prostate cancer, dynamic expression of SNAI2 could predict tumor progression and drug sensitivity (13). Furthermore, in gastric cancer, SNAI2 also plays an important role in tumor progression (14). In addition to the above tumors, SNAI2 was also involved in the formation and metastasis of other common tumors, including non-small cell lung cancer cells (15), and colorectal cancer (16). Also, SNAI2 plays a critical role in the epithelial-mesenchymal transition process (17, 18). Therefore, SNAI2 plays an indispensable role in a variety of tumors, which further increases our interest in pan-cancer analysis. Overexpression of SNAI2 is a common occurrence in human tumors, and it is associated with a bad prognosis in cancer patients (19–21). Overexpression of SNAI2 generated by stimulation TGF signaling gave cancer cells migratory and invasive characteristics (22). According to research, SNAI2 epigenetic silencing governs dynamic variations in SNAI2 expression, and restoring SNAI2 expression with panobinostat improves dasatinib sensitivity, indicating a new therapeutic strategy for prostate cancer (13). Furthermore, SNAI2 contributed to tumorigenicity and chemotherapy resistance in pancreatic cancer by regulating IGFBP2, however (23), however, the overall role of SNAI2 in pan cancer has not been analyzed yet

At present, pan-cancer analysis has become increasingly popular due to the continuous development and advancement across cancer kinds of multi-omics data, which also promoted a more comprehensive understanding of the pathogenesis of malignant tumors. Given the complexities of tumorigenesis, it is critical to perform a pan-cancer expression analysis of any gene of interest and assess its relationship to clinical prognosis and potential molecular mechanisms. However, the role of SNAI2 in cancer immune infiltrations and immunotherapy response prediction is not clear, and no comprehensive pan-cancer study has been performed yet. Therefore, in this study, our systematic and detailed analysis of SNAI2 in pan-cancer provides a broad elaboration of cancer biology by using The Cancer Genome Atlas (TCGA) (24), Cancer Cell Line Encyclopedia (CCLE) (25), and Genotype-Tissue Expression (GTEx) (26) databases. We appraised the aberrant expression levels of SNAI2 in pan-cancer compared to human normal tissues, and further examined the upregulated SNAI2 protein and mRNA expression levels in clinical PAAD samples. Besides, we also performed the genomic alteration analysis, prognosis analysis, gene set enrichment analysis (GSEA), and immune cell infiltration analysis of SNAI2 in pan-cancer, the most interesting finding is that SNAI2 is a robust prognostic biomarker for pan-cancer and predict the immunotherapy response effectively, and provided the main thread for further investigation on the role of SNAI2 in cancer immunity.

## Methods

### SNAI2 expression analysis in pan-cancer

To get a comprehensive analysis of SNAI2 expression patterns, we analyzed the SNAI2 levels in normal tissues and tumor cell lines according to the GTEx and CCLE databases. In the TCGA database, we used the Gene_DE module of TIMER2 (tumor immune estimation resource, version 2) web (http://timer.cistrome.org/) to investigate SNAI2 expression differences between tumors and neighboring normal tissues. For certain tumors without normal or with highly limited normal tissues, we used the GEPIA2 (Gene Expression Profiling Interactive Analysis, version 2) web server (27) to integrate and analyze the two databases (GTEx and TCGA databases).

### Immunohistochemistry and immunofluorescence analysis

IHC of SNAI2 was obtained from the THPA (https://www.proteinatlas.org) to assess the expression at the protein level in various types of cancers including pancreatic cancer. The antibody to SNAI2 for immunohistochemistry is CAB011671. Also, the survival analysis in SNAI2 immunohistochemistry was obtained from the aforementioned website. For Immunofluorescence (IF), the cells (HEK293T, Hela, HUVEC, and HCCLM3) were fixed with 4% paraformaldehyde and incubated with 0.1% TritonX-100, then stained with anti-SNAI2 (1:100) and phalloidin (1:1000) overnight at 4°C. After incubating secondary antibodies, the cell nuclei were counterstained with DAPI (1:1000). Finally, take photos with a fluorescence microscope.

### Analysis of gene mutation landscape

We analyzed the genetic alteration characteristics of SNAI2 in Pan-Cancer by logging into the cBioPortal web (28, 29) (https://www.cbioportal.org/). The Cancer Types Summary module showed the frequency of modification, mutation type, and CNA (Copy number alteration) across all TCGA tumors. The mutated site of SNAI2 was observed in the 3D structure. The TCGA database was used to gather data on the expression of MMR genes (MLH1, MSH2, MSH6, EPCAM, and PMS2) as well as DNA methylation surrogates in pan-cancer. For subsequent analysis, the expression data were log2 transformed.

### Prognosis analysis of SNAI2 in an-cancer

The UCSC Xena database (https://xenabrowser.net/datapages/) was used to assess the prognosis value of SNAI2 in terms of overall survival (OS), disease-specific survival (DSS), disease-free interval (DFI), and progression-free interval (DFI). Then univariate Cox regression and Kaplan-Meier model were used to assess the prognostic role of SNAI2 for a specific prognosis type in each cancer. Moreover, bivariate SNAI2 expression levels were used to perform Kaplan Meier curve analysis, whose cutoff was chosen by the surv-cutpoint function of the survminer R package (0.4.9) The log-rank p-value of the K-M method and hazard ratio (HR) with a 95% confidence interval was computed, and the outcomes were presented as a heatmap.

### Gene set enrichment analysis

To analyze the effect of SNAI2 expression on cancers, we conducted the GSEA computational method to explore SNAI2 enrichment. The “gmt” file of the hallmark gene set (h.all.v7.4.symbols.gmt), which contains 50 hallmarks gene sets, was downloaded from the website of Molecular Signatures Database (MSigDB, https://www.gsea-msigdb.org/gsea/index.jsp), and used to calculate the normalized enrichment score (NES) and false discovery rate (FDR) of the DEGs between low- and high-SNAI2 expression cancer group for each biological process in each cancer type. The GSEA was carried out with the help of the R package “clusterProfiler” (30) and the results were summarized in the bubble plot depicted by the R package “ggplot2”.

### Immune cell infiltration analysis in TIMER2

TIMER is a data source for quantifying immune cell infiltrations across distinct cancers. The immune cell infiltration levels of TCGA cancers were obtained from the TIMER2 database (http://timer.cistrome.org/). We investigated the relationship between SANI2 mRNA expression and 21 immune cell subsets by using the R package “ggplot2”, and generated a heatmap by Spearman correlation analysis.

### Single cell analysis of SNAI2

We using the Tumor Immune Single-cell Hub (TISCH) web tool (http://tisch.comp-genomics.org/documentation/) to explore the single-cell analysis (31). The analysis parameters were as below: SNAI2 (Gene), major-lineage (Cell-type annotation), and all cancers (Cancer type). The expression levels of SNAI2 in each cell type were quantified and visualized by a heatmap.

### Immunotherapy prediction analysis

The Spearman correlation analysis was performed to show the associations between SNAI2 and reported biomarkers of cancer immunotherapy for each cancer type. Tumor mutation burden (TMB) and microsatellite instability (MSI) were the well-known immunotherapy biomarkers. In this study, we analyzed the correlations between SNAI2 and immunotherapy biomarkers in pan-cancer by TCGA database. We first acquired the gene mutation data of all cancer types possessed from the TCGA database, then calculated the TMB and MSI of each cancer sample. Then, we analyzed the association between SNAI2 expression with 47 immune checkpoint-related genes, TMB, and MSI with Spearman’s correlation method. Moreover, the Sangerbox website (http://sangerbox.com) was utilized to investigate the correlation between SNAI2 expression and neoantigens *via* the “Tool” module and Spearman’s correlation test. All the results were visualized as heatmaps or radar plots. Furthermore, three immune checkpoint blockade (ICB) therapy cohorts were obtained to validate the immunotherapy response prediction ability of SNAI2. The GSE91061 cohort includes 51 melanoma patients receiving nivolumab (anti-PD-1). The Gide2019 cohort includes 32 melanoma patients with anti-PD-1 and anti-CTLA4 therapy. The IMvigor210 cohort contains 348 urological cancer patients treated with atezolizumab (anti-PDL1). Patients were divided into a SNAI2 low-expression group and a high-expression group according to the best cut-off value using the surv-cutpoint function of the “survminer” R package (0.4.9). A Chi-square test was used to evaluate the proportion difference of responders between low- and high-SNAI2 cancer groups.

### Cell culture, reagents, and RNA interference

The Chinese Academy of Sciences Shanghai cell bank provided human pancreatic cancer cells (PANC-1, SW1990, BxPC-1, SW-979) and an H6C7 cell line for this study. The cells were cultured in Dulbecco’s modified Eagle’s medium (DMEM) supplemented with 10% fetal bovine serum (Gibco, USA) (FBS) and 100 units/mL of penicillin-streptomycin (Invitrogen, USA). All cells were grown at 37°C with 5% CO_2_ in a humidified cell incubator. The antibodies and reagents were described in **Supplemental Table 1**. Plasmids encoding shRNAs against SNAI2 were synthesized by Sheweisi Biotechnology Company (Tianjin, China) In addition, the vector encoding SNAI2 was got from Genepharma Company. The cells were transfected with overexpression constructs or shRNA plasmids using Lipofectamine 3000 (Invitrogen, USA) according to the manufacturer’s instructions. Transfected cells were cultured in DMEM medium without FBS and replaced with a complete medium after 6-8h. Subsequent experiments were conducted after transfection for 48 h.

### Western blotting and quantitative real-time PCR

For the western blotting assay, equal amounts of cell lysates were separated using SDS/PAGE, transferred to polyvinylidene fluoride (Millipore, USA) membranes, and then blocked in 5% skim milk. Next, the electrotransferred membranes were incubated overnight at 4°C with the indicated primary antibodies and were incubated with appropriate HRP-conjugated anti-mouse/rabbit secondary antibodies for one hour at room temperature. Lastly, immunoreactive bands were visualized with chemiluminescence kits chemiluminescence after three washes with Tris-buffered saline with 0.1% Tween (TBST). To investigate the expression level of SNAI2 mRNA in tumor tissues and cells, we conducted a qRT-PCR experiment by the conventional method. Briefly, total RNA was isolated from cells or tissues using Trizol reagent (Invitrogen, USA) and qRT-PCR was conducted by using the SYBR Green qPCR Master Mix. The primers used in PCR are as followed: SNAI2, 5’-CGAACTGGACACACATACAGTG-3’ and 5’-CTGAGGATCTCTGGTTGTGGT-3’; GAPDH, 5’-AGAAGGCTGGGGCTCATTTG-3’ and 5’-AGGGGCCATCCACAGTCTTC-3’.

### Cell proliferation assays and colony formation assays

To explore the effect of SNAI2 on cell proliferation, we conducted CCK-8 and 5-ethynyl-2′-deoxyuridine (EdU) staining assays. For the CCK-8 assay, multiple cultures of cells were plated in 96-well plates at a density of 4.0×10^3^ cells/well. At the indicated time points, viable cells were assessed using the CCK-8 reagent according to the manufacturer’s instructions (KeyGEN, Shanghai, China). For EdU staining assays, the transfected cells were planted in the 96-well plate, incubated for 36 h, and conducted by using a Cell-Light EdU DNA Cell Proliferation Kit (Ribobio, Guangzhou, China), which was calculated as the ratio of the number of EdU-positive cells to the number of total cells. In the colony formation assay, the transfected cells were plated in 6-well plates at a density of 500 cells/well and cultured for 14 days. Then the plates were fixed and stained with 0.5% crystal violet.

### Cell migration and invasion assays

Transwell is a common detection method for cell migration, which refers to the movement of cells after receiving a migration signal or feeling the gradient of certain substances. The principle is to plant the cells in the upper chamber. Because the polycarbonate membrane is permeable, the components in the lower layer of the culture medium can affect the cells in the upper chamber. Cell mass can reflect the migration ability of tumor cells. Therefore, we used this experiment to explore the effect of changing SNAI2 on cell invasion and migration. Transwell systems (8-m pore size; BD Biosciences, USA) were used for migration and invasion assays. Briefly, 5×10^4^ cells resuspended in DMEM were seeded into the upper chambers not coated (for migration assay) or coated with Matrigel (for invasion assay; BD Biosciences, Franklin Lakes, USA). The lower chambers were filled with a 15% FBS medium. The chambers were removed after the indicated periods of incubation, and cells on the lower surface of the membrane were fixed, stained with 0.1% crystal violet, and photographed. For each chamber, five random visual fields were manually counted, and migration and invasion experiments were repeated three times independently.

### Statistical analysis

Paired student’s-tests were used to assess gene expression data from the TCGA and GTEx datasets. Spearman’s correlation was used to assess the relationships between SNIA2 expression and immune cell abundance scores. The Kaplan-Meier technique and univariate Cox regression analysis were employed to assess the prognostic value of SNAI2 expression in each malignancy. To compare the proportions of ICI-therapy responders and non-responder between low-SNAI2 and high-SNAI2 cancer subgroups, a chi-square test was used to compute the statistical significance. The experimental data were analyzed with GraphPad Prism 9.0 software. The differences between groups were analyzed by using Student’s *t*-test or one-way analysis of variance (ANOVA). All experiments were repeated in triplicates. The results were reported as the Mean ± SD. All statistical tests were two-sided, and statistical significance was set at *p <*0.05. The abbreviations of cancers were represented in **Supplemental Table 2**.

## Results

### SNAI2 expression analysis in human pan-cancer

**Figure 1:**
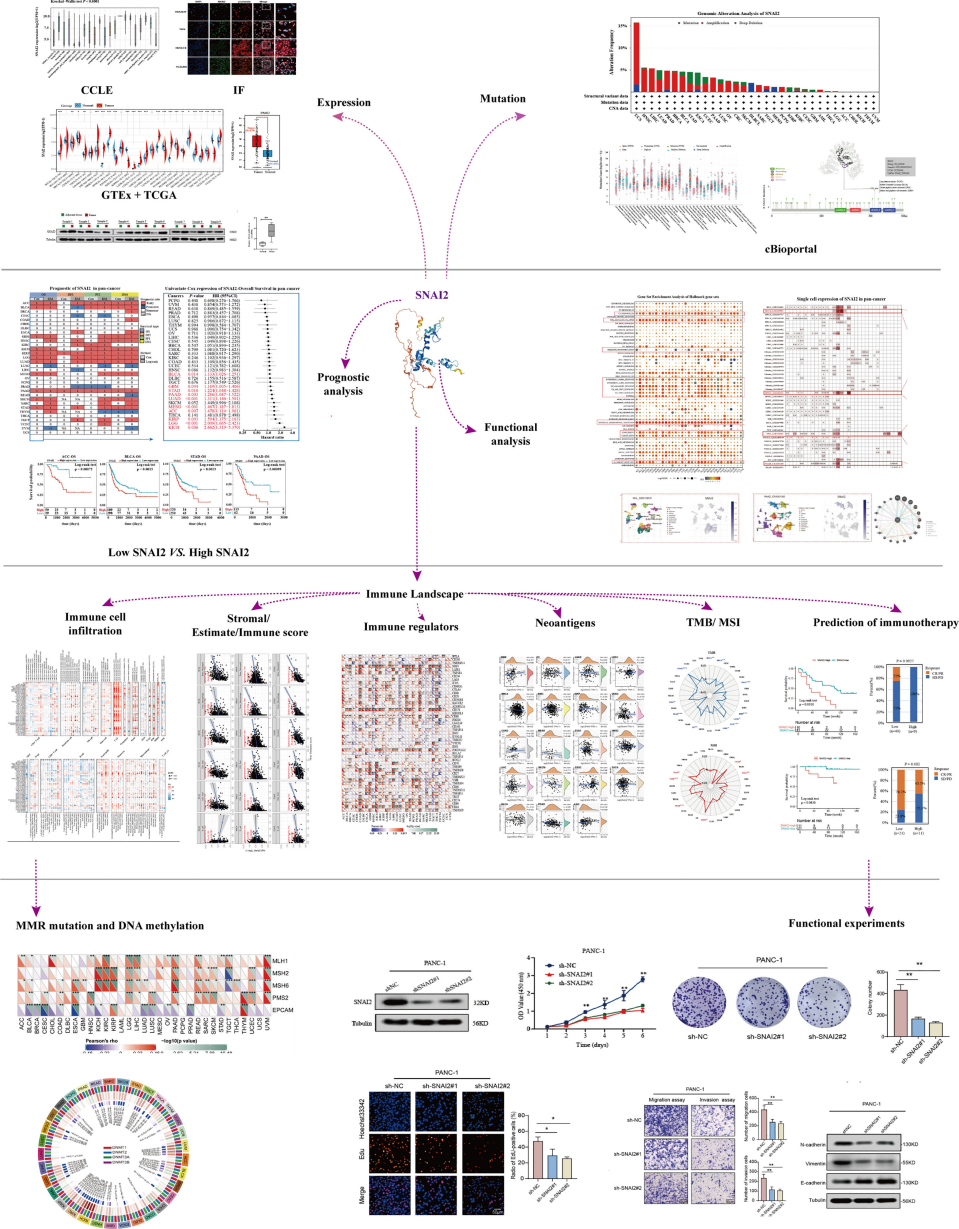
Design and workflow of this study. *p < 0.05, **p < 0.01, ***p < 0.001.

**Figure 2:**
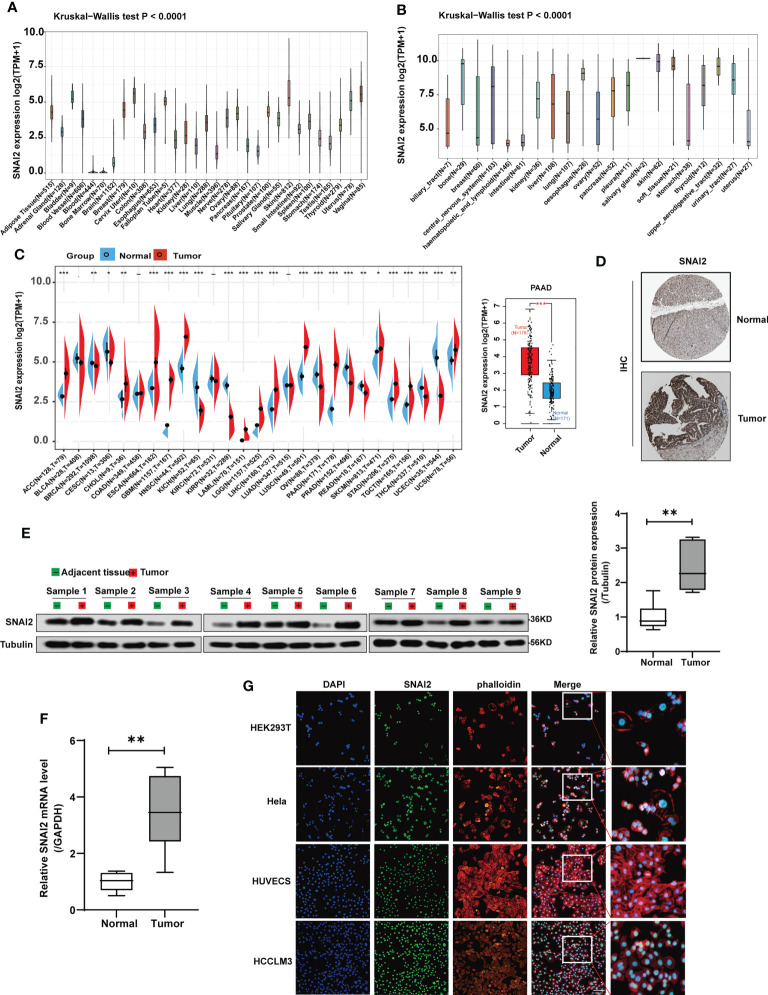
SNAI2 expression analysis in pan-cancer analysis. **(A)** The expression level of the SNAI2 in normal tissues. **(B)** The expression level of the SNAI2 in tumor cell lines. **(C)** The expression level of the SNAI2 between different tumors and normal tissues was analyzed based on TCGA and GTEx databases. The box plot data were supplied, and Log2 (TPM+1) was applied for the log scale. **(D)** Representative images of SNAI2 immunohistochemical staining analysis in the PAAD tissue and adjacent normal tissue in the HPA database. **(E)** Western blotting analysis of SNAI2 protein expression in the paired PAAD tissues and adjacent normal tissues. **(F)**The SNAI2 mRNA expression in 9 pairs of PAAD tissues and adjacent normal tissues was evaluated using qRT-PCR. **(G)** The immunofluorescence images showed the distribution of SNAI2 in the HEK293T, Hela, HUVEC, and HCCLM3 cell lines. All data are presented as the mean ± SD of three independent experiments. *p < 0.05, **p < 0.01, ***p < 0.001, ns, no significance.

In this paper, pan-cancer samples from public databases were included for subsequent analysis including expression differences, the landscape of SNAI2 mutations, the correlation between expression and survival, and immune infiltration. The flow chart of our study design is shown in **Figure 1**. Firstly, we use the GTEx database to analyze the SNAI2 expression in normal tissues. The expression of SNAI2 was shown to vary in different organs, with significantly lower expression in blood and bone marrow (**Figure 2A**). In addition, SNAI2 expression was highly expressed in most tumor cells according to the CCLE database (**Figure 2B**).

To determine the SNAI2 expression in cancers and their corresponding adjacent non-cancerous tissues, we employed the TCGA and GTEx datasets to analyze the SNAI2 mRNA levels. The results revealed that high SNAI2 expression was examined in some cancers including ACC, CHOL, ESCA, GBM, HNSC, LAML, LGG, LIHC, LUSC, PAAD, SKCM, STAD, TGCT, and UCS. By contrast, low SNAI2 expression was observed in BRCA, CESC, KICH, KIRP, OV, PRAD, READ, THCA, and UCEC (**Figure 2C**). Furthermore, IHC results also revealed that SNAI2 expression was located in the nuclear, SNAI2 protein was expressed to some extent in both normal pancreatic tissue and pancreatic cancer, and the staining level was significantly increased in pancreatic cancer (**Figure 2D**).

Therefore, we further performed western blot and qRT-PCR assays in clinical PAAD samples to confirm the expression of SNAI2, and the result indicated that the SNAI2 protein and mRNA were upregulated in PAAD samples as expected compared with that in normal tissues (**Figures 2E, F**). Lastly, immunofluorescence (IF) images showed that the SNAI2 protein was mainly localized and distributed in the nucleus in HEK293T, Hela, HUVEC, and HCCLM3 cell lines (**Figure 2G**). Therefore, the above results showed that SNAI2 was differentially expressed in various cancers, which suggested that SNAI2 may play an important role in cancer progression.

### Genetic alteration of SNAI2 analysis in pan-cancer

**Figure 3:**
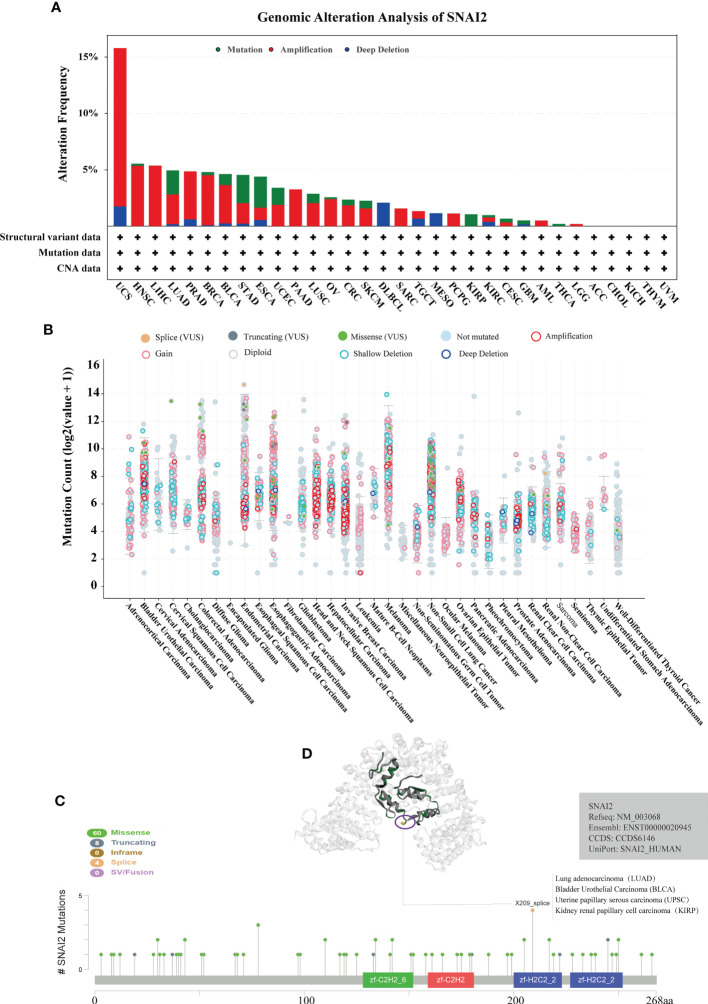
Analysis of mutation feature of SNAI2 in different tumors using TCGA database. **(A)** The alteration frequency with mutation type in the SNAI2 gene. **(B)** The mutation count of the SNA2 gene in various cancers. **(C)** The specific alteration site of the SNAI2 gene. **(D)** The 3D structure of SNAI2 in the mutation site with the highest alteration frequency (X209_splice) was displayed.

In order to explore the mutation rate and mutation type of SNAI2 in different tumors, the genetic alteration of SNAI2 in various cancers from the TCGA cohorts was analyzed by the cBioPortal tool. We found that the highest alteration frequency of SNAI2 (>15%) appears for patients with UCS, in which amplification was the primary alteration type. It is worth noting that the only type of SNAI2 gene alteration in Lymphoid Neoplasm Diffuse Large B-cell Lymphoma (DLBCL) and MESO cases was deep deletion. In addition, we also found that amplification was the most common mutation type, followed by mutation, and finally deep mutation. (**Figure 3A**). Also, the mutation count of SNAI2 in different cancers was shown in **Figure 3B**. Further, we also presented the types, sites, and case numbers of the SNAI2 genetic alteration. The main type of SNAI2 genetic alteration was missense mutation followed by truncating mutation (**Supplemental Table S3**). The X209_splice alteration was located in the zf-H2C2_2 domain of SNAI2, which can induce a splice mutation of the *SNAI2* gene, which further leads to related functional abnormalities, mainly including the gain in BLCA, diploid in UCEC and KIRP, shallowdel in LUAD (**Figure 3C**). We also presented the 3D structure of the X209_splice mutation in the SNAI2 gene (**Figure 3D**).

### Prognostic significance of SNAI2 in pan-cancer

**Figure 4:**
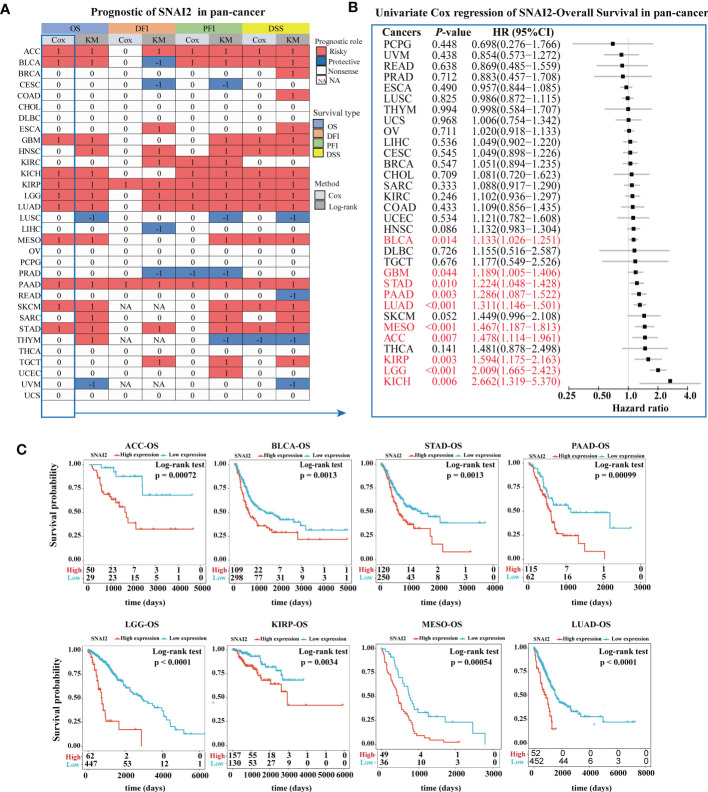
Prognostic Value of SNAI2. **(A)** Summary of the correlation between expression of SNAI2 with overall survival (OS), disease-specific survival (DSS), disease-free interval (DFI), and progression-free interval (PFI) based on the univariate Cox regression and Kaplan-Meier models. Red indicated that SNAI2 was a risk factor affecting the prognosis of cancer patients, and blue represents a protective factor. Only *p* values < 0.05 were shown. **(B)** Univariate Cox regression analysis of SNAI2 in pan-cancer (OS). **(C)** Kaplan-Meier overall survival curves of SNAI2 in ACC, BLCA, STAD, PAAD, LGG, KIRP, MESO, and LUAD.

The prognostic significance of SNAI2 in various cancers was evaluated (**Figure 4A**). According to the results of Kaplan-Meier OS analysis, SNAI2 is a risk factor for ACC, BLCA, GBM, HNSC, KICH, KIRP, LGG, LUAD, MESO, PAAD, SKCM, SARC, STAD, THYM, and a protective factor in LUSC, and UVM. The results shown in the forest plot (**Figure 4B**) demonstrated that the downregulating of SNAI2 expression was related to the time delay of OS in ACC, BLCA, GBM, KICH, KIRP, LGG, LUAD, MESO, PAAD, and STAD. Furthermore, we analyzed the Kaplan-Meier curve of ACC, BLCA, STAD, PAAD, LGG, KIRP, MESO, and LUAD, and the results suggested that lower expression of SNAI2 was related to a better survival outcome (**Figure 4C**), which indicates that SNAI2 was a prognostic biomarker in these cancers. Therefore, SNAI2 had a prognostic role in predicting the prognosis of cancers, but the roles were complicated and multifaceted across cancers. Further investigation should focus on the function of the SNAI2 protein in cancer cells.

### Gene set enrichment analysis in pan-cancer

**Figure 5:**
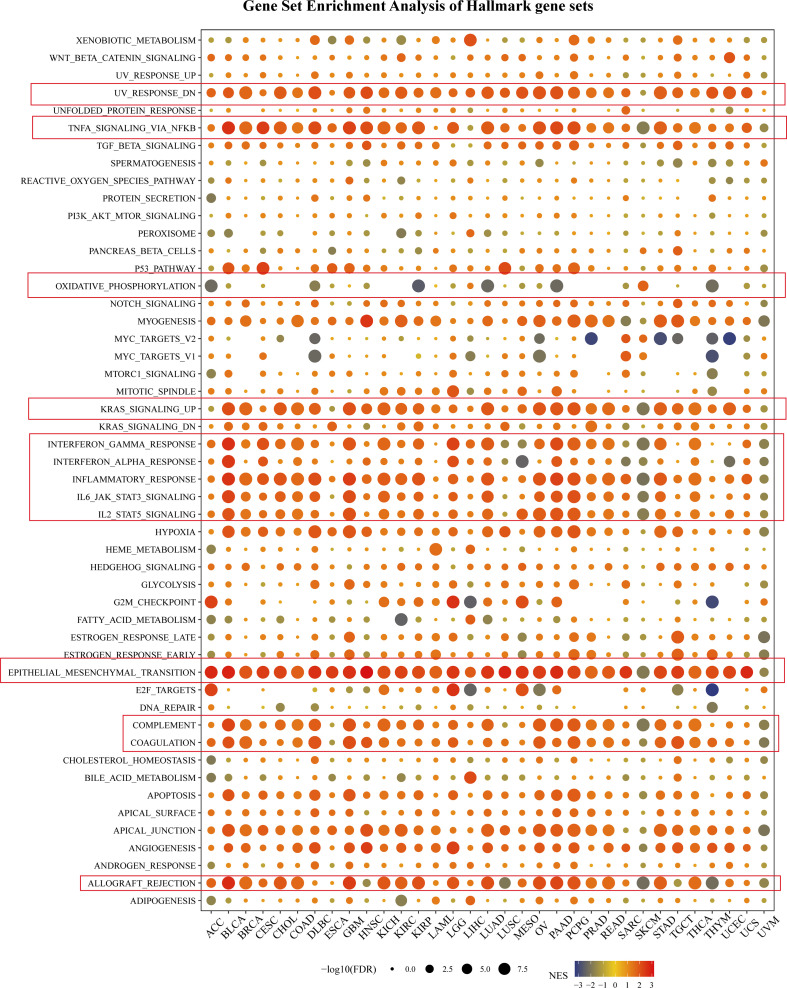
Gene Set Enrichment Analysis of Hallmark gene sets. The size of the circle represented the FDR value of the enriching term in each cancer, and the color indicated the normalized enrichment score (NES) of each term.

To further explore the tumorigenic role of SNAI2, the differential expression genes (DEGs) between low- and high-SNAI2 subgroups in each cancer were used to perform GSEA to discern the SNAI2-associated cancer hallmarks (**Figure 5**). It is found that SNAI2 expression was significantly related to immune-related pathways, such as UV response (down), TNFA-signaling-via-NFKB, KRAS signaling (up), IFN-α response, IFN-γ response, inflammatory response, IL-JAK-STAT3 signaling, IL2- STAT5 signaling, complement, coagulation, and allograft rejection, especially in BLCA, COAD, GBM, PAAD, PCPG, and STAD. It is noteworthy that the expression of SNAI2 is significantly negatively correlated with oxidative phosphorylation in the ACC, KIRP, LUAD, PAAD, and THYM. These data uncovered a potential association between SNAI2 expression and immune activation in the tumor microenvironment (TME).

In addition, the epithelial-mesenchymal transition (EMT) hallmark of many kinds of tumors and had a significant positive correlation with ACC, BLCA, BRCA, CESC, CHOL, COAD, DLBC, ESCA, GBM, HNSC, KICH, KIRC, KIRP, LAML, LGG, LUAD, LUSC, MESO, OV, PAAD, PCPG, PRAD, READ, SKCM, STAD, TGCT, THCA, THYM, UCEC, and UCS. In previous studies, EMT has been significantly confirmed to be related to the occurrence, metastasis, and drug resistance of cancers (32), suggesting that SNAI2 might play an indispensable part in the oncogenesis and development of cancers by enrolling in EMT. The above results provided an important direction for further understanding the role of SNAI2 in cancer establishment and development.

### Single cell analysis of SNAI2 in cancers

**Figure 6:**
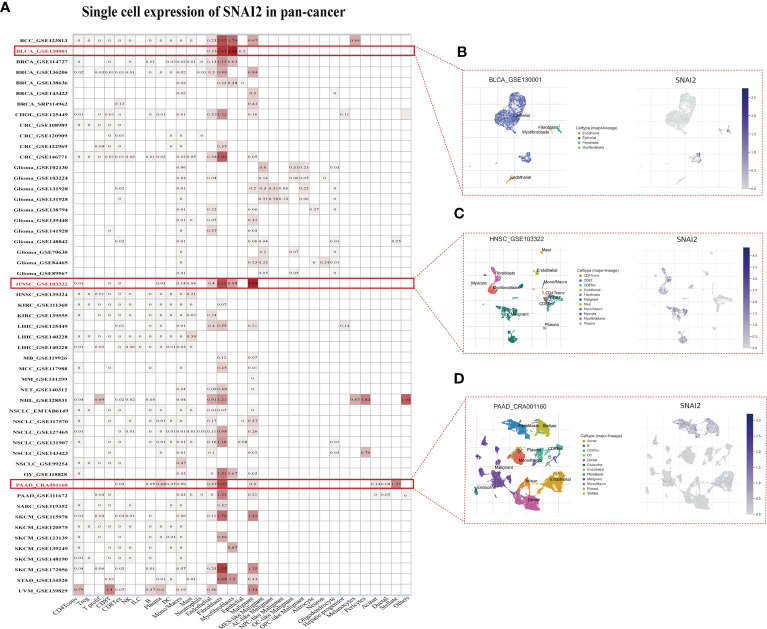
Single-Cell Analysis of SNAI2 in Cancers **(A)** Summary of SNAI2 expression of various cell types in single-cell datasets. **(B)** The Scatter plot showed the distributions of 4 different cell types of the GSE130001 BLCA dataset and the SNAI2 expression levels of cells in the GSE130001 dataset. **(C)** The Scatter plot showed the distributions of 11 different cell types of the GSE103322 HNSC dataset and the SNAI2 expression levels of cells in the GSE103322 dataset. **(D)** The Scatter plot showed the distributions of 12 different cell types of the CRA001160 PAAD dataset and the SNAI2 expression levels of cells in the CRA001160 dataset.

The single-cell analysis of SNAI2 in single-cell datasets of cancer samples was analyzed. The heatmap depicted (**Figure 6A**) represents the expression levels of SNAI2 of various cell types including immune cells, stromal cells, malignant cells, and functional cells. And the finding revealed that SNAI2 was mainly expressed in the fibroblasts, especially in BCC, BLCA, CRC, HNSC, PAAD, SKCM, and STAD cell datasets. In the GSE130001 BLCA dataset, the SNAI2 is highly expressed in fibroblasts and myofibroblasts cells in the BLCA microenvironment (**Figure 6B**). In the GSE103322 HNSC dataset, the SNAI2 is highly expressed in fibroblasts and malignant cells in the HNSC microenvironment (**Figure 6C**). In the CRA001160 PAAD dataset, SNAI2 was highly expressed in fibroblast cells and stellate cells in the PAAD microenvironment (**Figure 6D**).

### TIMER immune cell infiltration analyses

**Figure 7:**
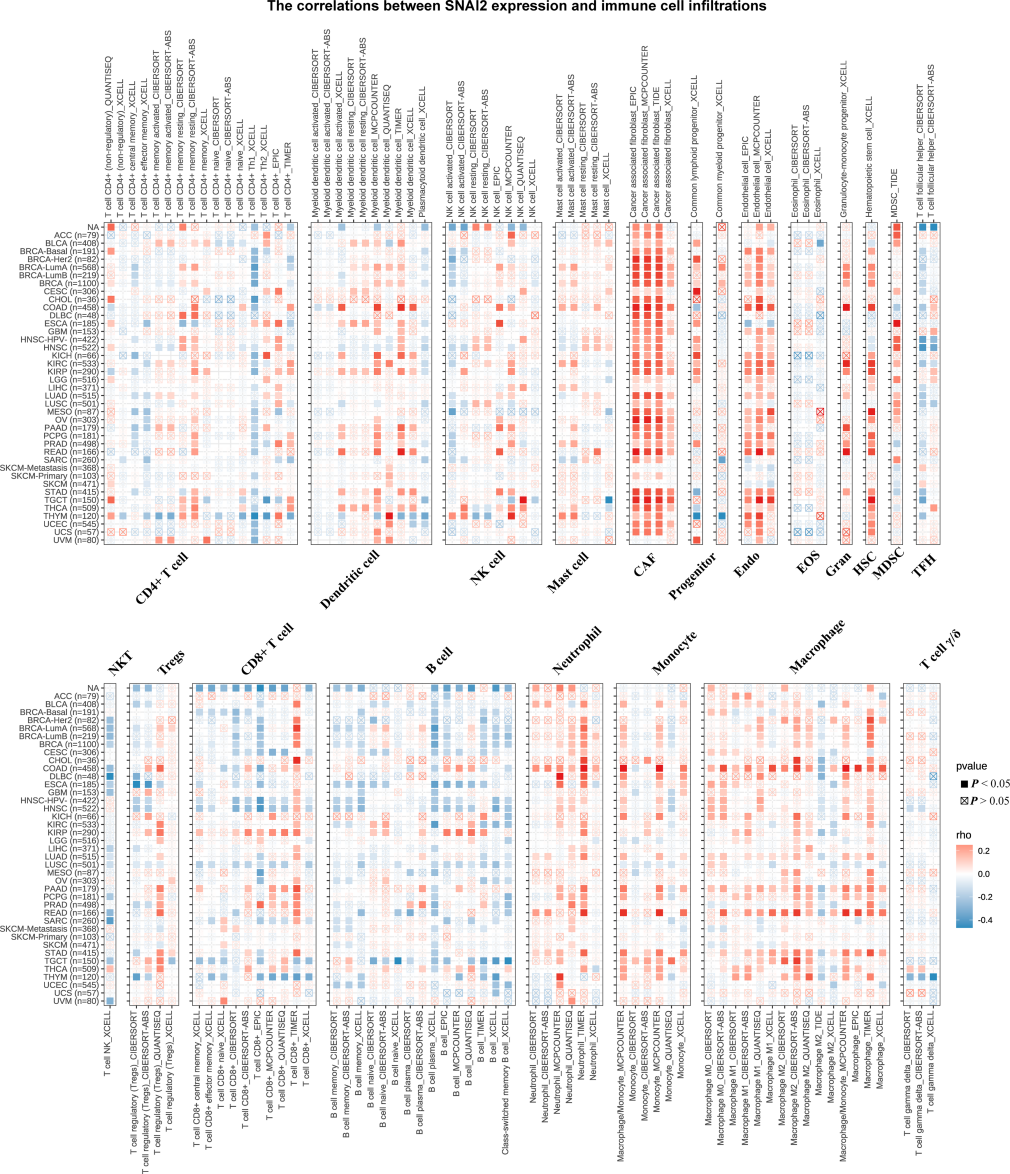
Immune Cell Infiltration Analyses. The relationship between SNAI2 expression and the levels of infiltration of CD4+ T cells, CAF, progenitor cells, Endo, Eos, HSC, Tfh, gdT, NKT, regulatory T cells (Tregs), B cells, neutrophils, monocytes, macrophages, dendritic cells, NK cells, Mast cells, and CD8+ T cells in cancer. A positive correlation was shown in red, while a negative correlation was shown in blue.

The correlations between SNAI2 expression and immune cell infiltrations were analyzed to further elucidate the relationships between SNAI2 and cancer immunity. Spearman correlation analyses were conducted utilizing pan-cancer immune cell infiltration data from the TIMER2 database. As shown in **Figure 7**, SNAI2 was positively associated with the infiltration levels of CAF, Endo, Neutrophil, Monocyte, Macrophage, Gran, and HSC, and negatively associated with the infiltration levels of B cell plasma in most TCGA cancers, especially in BRCA, HNSC, TGCT. In addition, the infiltration levels of NK T cells were also negatively associated with SNAI2 in DLBC, SARC, and UVM. We then integrated ImmuneScore, EstimateScore, StromalScore, and neoantigens to further evaluate the relationship between SNAI2 expression and immune infiltration across cancers. According to the results, SNAI2 expression was positively correlated with the ImmuneScore, EstimateScore, and StromalScore in most cancers (**Figures S1**–**S3**). Also, SNAI2 expression positively correlated with the neoantigens in GBM, while was negative in PRAD (**Figure S4**). Together, our results indicated that SNAI2 might affect the development, prognosis, and therapy of cancers by associating with immune cells.

### Relationships between SNAI2 and immune regulators, TMB, and MSI in cancers

**Figure 8:**
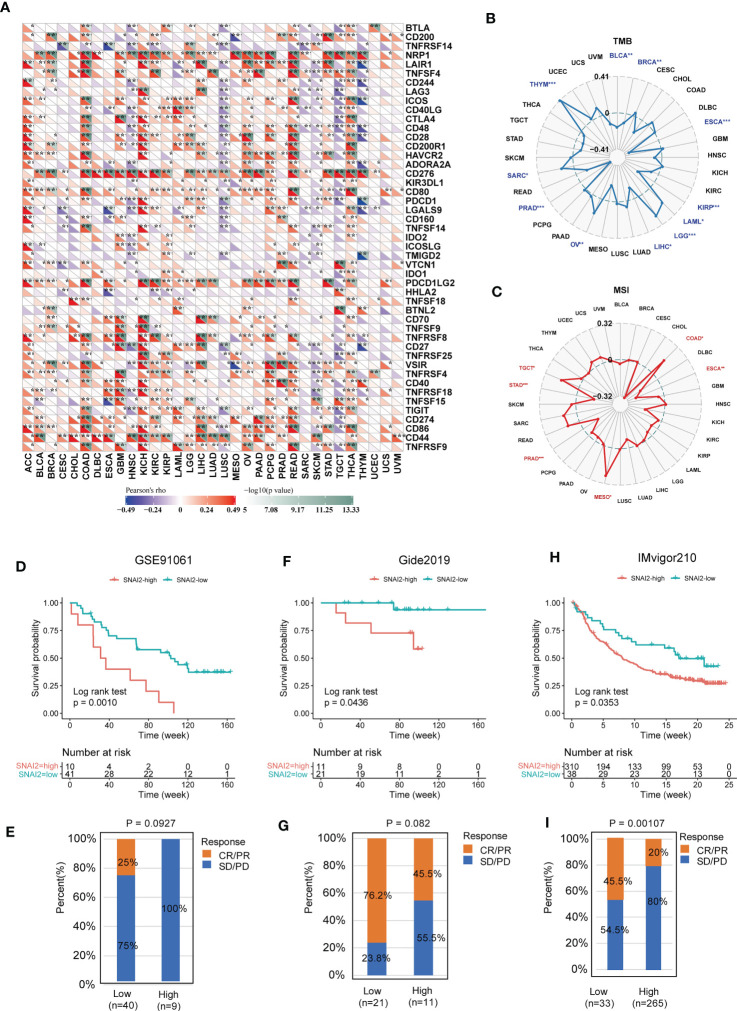
Immunotherapy prediction analysis of SNAI2 in the pan-cancer. **(A)** The correlation between SNAI2 expression and immune checkpoint gene expression in cancers. **(B)** The radar chart displayed the correlation between SNAI2 expression and TMB. **(C)** The radar chart displayed the correlation between SNAI2 expression and MSI. **(D)** Kaplan Meier curves for low-SNAI2 and high-SNAI2 expression from the GSE91061 clinical cohort (anti-PD-1 immunotherapy), and **(E)** the fraction of melanoma patients with response to the blockade in the two groups. **(F)** Kaplan Meier curves for low-SNAI2 and high-SNAI2 expression from the Gide2019 clinical cohort (anti-PD-1 and anti-CTLA4 immunotherapy), and **(G)** the fraction of melanoma patients with response to the blockade. **(H)** Kaplan Meier curves for low-SNAI2 and high-SNAI2 expression from IMvigor210 clinical cohort (anti-PD-L1 immunotherapy), and **(I)** the fraction of urological tumors with therapeutic response to the blockade. *p <0.05, **p <0.01, ***p < 0.001.

The associations between SNAI2 and immune regulators in pan-cancer were displayed (**Figure 8A**). We found that SNAI2 had a strong positive relationship with most immune regulators in ACC, COAD, KICH, READ, and THCA, and a negative relationship with most immune regulators in HNSC, LUSC, SKCM, and TGCT. Besides, there was a robust positive relationship between SNAI2 and NRP1, and CD276 in most of the TCGA cancers. Previous studies find the correlation between PDIA3 and NRP1, CD276 was also very significant in pan-cancer (33). To understand the role of SNAI2 in predicting the efficiency of immune checkpoint inhibitors (ICIs), the correlation between SNAI2 expression and TMB and MIS was further assessed. Positive correlations with TMB were identified in LAML, LGG, OV, SARC, and THYM, and negative correlations were discovered in BLCA, BRCA, ESCA, KIRP, LIHC, and PRAD (**Figure 8B**). Moreover, in the correlation between SNAI2 expression and MSI, positive associations were discovered in COAD, MESO, and TGCT, and negative correlations were discovered in ESCA, PRAD, and STAD (**Figure 8C**). Our results suggested that SNAI2 had the potential to predict the efficiency of ICIs in the corresponding cancers.

### The correlation between SNAI2 expression and immunotherapy response

The researchers discovered a link between SNAI2 expression and immune cell infiltration, TMB, and MSI, suggesting a degree of correlation with immunotherapy efficacy. Therefore, we assessed the possible correlation between SNAI2 expression levels and immunotherapy response by analyzing previously reported clinical study cohorts. We discovered that patients with low SNAI2 expression had more clinical benefits and therapeutic responses to PD-L1 blockade therapy. In the GSE91061 melanoma cohort (**Figure 8D**), melanoma patients with SNAI2 low-expression had much better survival probability than patients with SNAI2 high-expression patients, and the response rate to anti-PD-1 was 0% in the SNAI2 high-expression subgroup, while 25% of patients responded to the anti-PD1 therapy in the SNAI2 low-expression subgroup (**Figure 8E**). Moreover, in melanoma tumors of the Gide2019 cohort, clinical advantages and therapeutic responses to PD-L1 and anti-CTLA4 blocking therapy were greater in patients with low SNAI2 expression (**Figure 8F**). And the response rate to anti-PD-L1 and anti-CTLA4 therapy was 76.2% in SNAI2 low-expression patients, which is significantly higher than 45.5% in SNAI2 high-expression patients (**Figure 8G**). Furthermore, similar results were found in melanoma patients treated with anti-PD-1 therapy. In urological tumors of the IMvigor210 cohort (**Figure 8H**), the response rate to anti-PD-L1 therapy was 20% in the SNAI2 high-expression patients, which is significantly lower than 45.5% in SNAI2 low-expression patients (**Figure 8I**). These findings supported the potential of SNAI2 for immunotherapy response prediction, indicating that it was a promising biomarker for cancer immunotherapy.

### SNAI2 is related to the expression of the MMR gene and DNA methylation in cancers

DNA mismatch repair (MMR) is a highly conserved biological mechanism that is critical for preserving genomic integrity (34). The deletion or mutation of key genes in MMR leads to abnormal DNA replication, which also promotes the occurrence and development of tumors (35). To assess the impact of SNAI2 in tumorigenesis, the correlation between SNAI2 expression levels and mutations in five MMR genes including MLH1, MSH2, MSH6, PMS2, and EPCAM was analyzed. According to the findings, the MMR genes were correlated with SNAI2, and this correlation was found in the majority of cancer types (**Figure S5A**). Especially in the UVM, SNAI2 expression was significant positive related to MLH1, MSH2, MSH6, and PMS2 genes. While, a negative correlation with the EPCAM gene was observed in BLCA, BRCA, CESC, COAD, ESCA, HNSC, KICH, LUAD, LUSC, OV, PAAD, PRAD, STAD, and UCEC.

DNA methylation affects a variety of genetic changes, including chromosomal structure, DNA stability, and DNA interaction with specific proteins. The abnormal state of DNA methylation is another important reason for promoting tumorigenesis, which has been heralded as a promising biomarker for diagnosis, treatment, and prognosis (36). The relationship between the expression of four methyltransferase genes (DNMT1, (DNMT2, DNMT3A, DNMT3B) and SNAI2 in pan-cancer was investigated further (**Figure S5B**). The result suggested that SNAI2 expression was closed correlated with at least one methyltransferases gene in SKCM, TGCT, THCA, THYM, UCEC, UVM, BLCA, BRCA, CESC, COAD, ESCA, GBM, HNSC, KICH, KIRC, KIRP, LGG, MESO, PAAD, PRAD, READ, and SARC. Therefore, our study found that SNAI2 might play a potential role in affecting MMR regulation and DNA methylation in various cancers.

### Enrichment analysis of SNAI2-related partners

**Figure 9:**
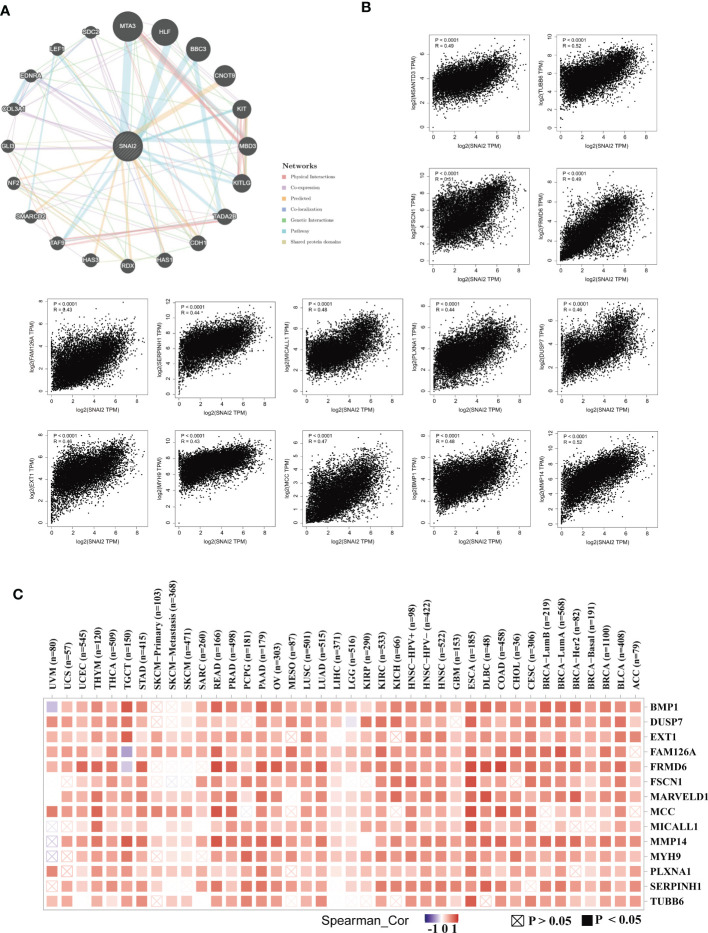
SNAI2-related gene enrichment analysis. **(A)** The protein-protein interaction (PPI) network presented the proteins interacting with SNAI2. **(B)** We also retrieved the top 100 SNAI2-linked genes in TCGA projects and evaluated the expression correlation between SNAI2 and chosen targeting genes, such as BMP1, DUSP7, EXT1, FAM126A, FRMD6, FSCN1, MARVELD1, MCC, MICALL1, MMP14, MYH9, PLXNA1, SERPINH1, SERPINH1, and TUBB6 using the GEPIA2 approach. **(C)** The corresponding heatmap data is provided in the detailed cancer kinds.

Next, we utilized the GeneMANIA online program to create a PPI network for SNAI2, and the SNAI2 expression-correlated genes for a series of pathway enrichment analyses to learn more about the molecular mechanism of the SNAI2 gene in carcinogenesis. SNAI2 demonstrated significant interactions with MTA3, HLF, BBC3, and CNOT9, as illustrated in **Figure 9A**. In addition, we tried to screen out the targeting SNAI2-binding proteins We combined the TCGA tumor expression data with the GEPIA2 program to find the top 100 genes that are linked with SNAI2 expression. The heatmap data revealed a positive correlation between SNAI2 and the fourteen genes in the majority of detailed cancer types (**Figures 9B, C**). SNAI2 expression levels were positively correlated with BMP1, DUSP7, EXT1, MMP14, TUBB6, MARVELD1, MCC, MICALL1, FSCN1, FRMD6, SERPINH1, PLXNA1, MYH9, and FAM126A.

### SNAI2 promote pancreatic cancer cells proliferation and invasion

**Figure 10:**
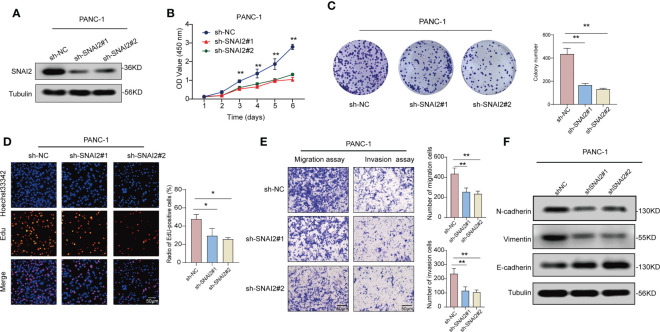
Knockdown of SNAI2 inhibits cell proliferation and promotes cell apoptosis of pancreatic cancer cells. **(A)** The knockdown efficiency of shSNAI2 was examined in PANC-1 cells with western blotting. **(B)** CCK-8 assays evaluated cellular growth curves in PANC-1 cells. **(C)** Representative images and quantification of colony formation assays of pancreatic cancer cells transfected with shSNAI2. **(D)** Representative images and quantification of EdU assays to evaluate cell proliferation ability after transfecting shSNAI2, magnification, ×200; scale bars, 50 µm. **(E).** Representative images and quantification of transwell assay to examine the invasion ability, magnification, ×200; scale bars, 50 µm. **(F)**. Western blotting showed the changes of EMT proteins in PANC-1 cells transfected with shSNAI2 plasmids. All data are presented as the mean ± SD of three independent experiments. *p < 0.05, **p < 0.01, ns, no significance.

To reveal the role of SNAI2 in pancreatic cancer cells cell proliferation and invasion, we designed the following assays. qRT-PCR and Western blot analysis suggested that SNAI2 was overexpressed in the four pancreatic cancer cell lines (PANC-1, SW1990, BxPC-1, and SW979) when compared with its expression in the control (**Figure S6A**). Firstly, we used the western blot assay to identify the knockdown status of SNAI2 in the PANC-1 cells. The result showed that SNAI2 shRNA could significantly knock down the protein level of SNAI2 (**Figure 10A**). To uncover the potential ability of SNAI2 in regulating PANC-1 cell proliferation and invasion, we performed the proliferation and transwell assays to reflect the proliferative and invasive ability of PANC-1 cells. CCK-8 (**Figure 10B**), colony formation (**Figure 10C**), and Edu (**Figure 10D**) analysis indicated that the proliferative ability of PANC-1 cells significantly decreased after SNAI2 was knocked down. Besides, transwell assays (**Figure 10E**) also proved that the knockdown of SNAI2 significantly weakened the invasive ability of PANC-1 cells. Conversely, overexpression of SNAI2 in SW1990 cells significantly promoted cell proliferation, migration, and invasion (**Figures S6B**–**F**). Moreover, EMT has been identified as an important process in tumor invasion and metastasis. We then used Western blot assay to look at the EMT markers (E-cadherin, N-cadherin, and vimentin) to see if SNAI2 could affect EMT in pancreatic cancer cells. The results showed that downregulating SNAI2 reduced the expression of N-cadherin and Vimentin while increasing E-cadherin expression in PANC-1 cells (**Figure 10F**), whereas overexpression of SNAI2 increased the expression of N-cadherin and Vimentin, and decreased E-cadherin expression in SW1990 cells (**Figure S6G**). Taken together, these data indicated that SNAI2 played a crucial role in pancreatic cancer cell proliferation and invasion.

## Discussion

Immune checkpoint blockade therapy has become one of the most important immunotherapies in cancer treatment in recent years and has transformed the landscape of cancer treatment (37), which also makes cancer patients see hope for treatment. Immune checkpoint blocking therapy promotes a long-lasting anti-cancer response by releasing a block in the immune system including include anti-PD-1, anti-PD-L1, and anti-CTLA4 therapy (38–40). However, due to the heterogeneity of each patient’s tumor suppressor microenvironment, most patients do not respond well to immunotherapy, and only a small percentage of cancer patients benefit from immunotherapy. In this study, we conducted a systematic analysis of SNAI2 to demonstrate the different roles in various cancers. And it is found that SNAI2 is a powerful pan-cancer prognostic biomarker, which can effectively predict immunotherapy response. As a result, our findings could help researchers learn more about the potential function of SNAI2 in cancer immunotherapy.

Firstly, we analyzed the SNAI2 expression in various cancers and cancer cell lines. SNAI2 was expressed differently in different human tissues and tumor cell lines. And according to the TGCA database and GTEx database, compared to the normal tissues, the SNAI2 expression level was decreased in CESC, KICH, KIRP, OV, PRAD, THCA, and UCEC, while increased in DLBC, GBM, HNSC, LUSC, PAAD, SARC, and THYM. The varied amounts of SNAI2 expression in various tumor types could indicate different underlying functions and processes. And problem worth discussing is that the survival probability was significantly decreased in ACC, BLCA, GBM, KICH, KIRP, LGG, LUAD, MESO, PAAD, SKCM, and STAD patients with the high-expression SNAI2 compared to the low-expression patients. We also conducted a clinical sample test with PAAD samples, and the results of western blot and qRT-PCR analyses indicated that the expression of SNAI2 in the PAAD tissues was remarkably higher than that in normal tissues adjacent to cancer, which was also consistent with the database results. Moreover, Kenji Masuo et al. also suggested that SNAI2 expression was overexpressed and it may represent an effective therapeutic target for pancreatic cancer by regulating IGFBP2 (23), which further supports the results of this analysis. In conclusion, these findings showed that SNAI2 was abnormally expressed in a variety of malignancies, and could be a new cancer biomarker. In addition, the analysis of genetic alterations in a pan-cancer cohort showed that the frequency of alterations in the SNAI2 gene was at a maximum of 15%. However, the types of mutations observed were non-specific and unlikely to have a significant impact on the development of cancer. Therefore, the abnormal expression of SNAI2 in tumor tissue cannot currently be associated with genetic alteration. Future studies could focus on exploring these possibilities and investigating the mechanisms underlying the dysregulation of SNAI2 in tumors. Secondly, the data from the SNAI2 gene survival prognosis analysis suggested different conclusions for different tumors. Results from OS, DSS, DFI, and PFI analyses were highly consistent, showing that SNAI2 is significantly associated with the prognosis of cancer patients. SNA2I was a risk factor for a large proportion of cancers, high-SNAI2 expression was significantly correlated with the poor prognosis of cancer patients including ACC, COAD, ESCA, GBM, HNSC, KIRC, KICH, KIRP, LGG, LUAD, MESO, PAAD, SKCM, SARC, STAD, TGCT, UCEC. While SNAI2 also acted as a protective factor for some types of cancers including CESC, LUSC, PRAD, READ, THYM, and UVM. Therefore, the above results showed that SNAI2 played an important role in predicting the prognosis and survival of cancer patients, and had the potential to become a powerful prognostic biomarker for cancer patients.

The GSEA result suggested SNAI2 was closely associated with immune-activated processes, such as KRAS signaling, IFN-α response, IFN-γ response, IL-JAK-STAT3 signaling, IL2-STAT5 signaling, inflammatory response, and EMT. These processes were most significantly enriched in high-SNAI2 cancer subgroups, but reverse results were found in ESCA, LAML, LUSC, SARC, and UCS. Previous studies have shown that the IL-6-JAK-STAT3 signaling pathway is abnormally over-activated in patients with hematopoietic malignancies or solid tumors, to encourage tumor cell proliferation, survival, invasion, and metastasis (41). And numerous studies have confirmed that EMT promoted cancer progression and metastasis (42–44). Combined with our immune pathway analysis, SNAI2 might play different regulatory roles in different tumors.

Another essential discovery of our study was that the expression of SNAI2 was highly related to immune infiltration in pan-cancer. Recent research had broadened our understanding of tumor biology to include the TME rather than just cancer cells. Immune and stromal cells, as important components of TME, played an important role in the initiation of human malignancies (45, 46). SNAI2 was associated positively with the degree of infiltration of CAF, Endo, Gran, HSC, Neutrophil, monocyte, and macrophage in most cancers, which suggested SNAI2 was most likely to affect the development and prognosis of cancers by shaping the tumor microenvironment. Activated fibroblasts, known as CAFs, exhibit a high degree of heterogeneity and are implicated in tumor progression and malignancy due to their activity in the stromal environment (47). The tumor microenvironment is largely composed of fibroblasts, and there is extensive evidence indicating that they can facilitate cancer development by promoting paracrine effects and assisting tumor cells through all stages of carcinogenesis (48). Furthermore, depletion of SNAI2 in CAFs results in reduced production of key tumorigenic factors such as SDF1, CXCL1, and MMPs (49). It was reported that SNAI2 plays a crucial role in SDF1 production by CAFs in both mice and humans, as well as in establishing the heterocellular signaling loop between cancer cells and fibroblasts (50). Therefore, our results suggested that the expression of SNAI2 in CAFs stimulates tumor cell migration thought the release of different cytokines into the medium to a certain degree, which is consistent with the results of Zhang et al. (50). Besides, the correlation analysis of SNAI2 and immune regulators in pan-cancer suggested that SNAI2 expression was correlated with many immune regulator gene expressions, especially in ACC, COAD, KICH, and READ. Furthermore, Zhou et al. found that B7 homolog 3 protein (B7-H3, also known as CD276), a newly identified immunoregulatory protein member of the B7 family, is an attractive and promising target for cancer immunotherapy (51). CD44, as a prognostic marker, is of great significance for clinical diagnosis and cancer treatment (52), and Neuropilin-1(NRP1) is a checkpoint target with unique implications for cancer immunology and immunotherapy (53). Combined with our finding that SNAI2 was strongly related to the expression of CD276, NRP1, and CD44 in many cancer types, we speculated that the potential association between SNAI2 and CD276, CD44, or NRP1 was promising for our further studies.

We further analyzed the expression level of SNAI2 and response to anti-PD-L1 immunotherapy in urological tumors, and anti-PD1 and anti-CTLA4 in melanomas. The results showed that SNAI2 was a powerful prognostic biomarker in urological tumors and can effectively predict the response to anti-PD-L1 immunotherapy, and consistent results were discovered in melanoma patients treated with anti-PD-1 and anti-CTLA4 therapy. Our findings indicate that SNAI2 was an effective biomarker for predicting response to immune checkpoint blockade therapy. Therefore, we believed that SNAI2 could be a robust immunotherapy biomarker for cancers, and it possessed the strong potential to be applied in clinical cancer treatment.

TMB and MSI, in addition to tumor PD-L1, PD-1, and CTLA4 expression levels, had emerged as potential biomarker possibilities. Higher TMB, according to Samstein et al, was linked to more tumor neoantigens, which could aid immune detection and boost anti-tumor immune responses (54), MSI was a predictive factor for the treatment outcome of many cancers, such as gastroesophageal adenocarcinoma (55), advanced gastric cancer (56). In total, the substantial relationship between SNAI2 and TMB, and MSI in tumor samples was demonstrated in this investigation. The method by which SNAI2 affected the expression of each TMB and MSI, however, had to be investigated further.

Tumorigenesis is mostly caused by gene mutations. In normal cells, the MMR gene mutation destroys the stability and integrity of the entire genome (57). DNA methylation was a type of chemical alteration of DNA that changes gene expression without affecting the sequence of the DNA (58). The abnormal changes of MMR and DNA methylation D are closely related to the occurrence and development of tumors. The findings of this study revealed that SNAI2 expression in various malignancies was linked to MMR gene and DNA methylation levels, implying that SNAI2 had an important role in tumorigenesis at both the genetic and epigenetic levels.

SNAI2 had been identified as an oncogene that plays a broad effect on cancer progression and metastasis in some cancers, such as ovarian cancer, breast cancer, and colorectal cancer (50, 59–61).In breast cancer, Fan et al. found that ASB13 inhibits breast cancer metastasis by promoting SNAI2 degradation and relieving its transcriptional repression of YAP (62). In this study, we focused on exploring the role of SNAI2 in pancreatic cancer progression using cell culture approaches and cell-based tumor functional models, and a series of functional experiments discovered that downregulating SNAI2 significantly inhibited the cell proliferation, invasion, migration abilities, and EMT in pancreatic cancer. Furthermore, Masuo et al. found that SNAIL2 contributes to tumorigenicity and chemotherapy resistance in pancreatic cancer by regulating IGFBP2 (23), which supported our research results to some extent. Nevertheless, the other associated mechanisms require further elucidation.

We explored different aspects of SNAI2 in this pan-cancer study and discovered that SNAI2 could be a powerful biomarker across cancer types, particularly in the era of immunotherapy. However, there are still some limitations. First, the results of this pan-cancer analysis are mainly derived from an integrated analysis of multiple databases. Due to the limited analysis method, there may be some systematic errors in this study. Second, this study presents the role of SNAI2 in various cancers through bioinformatics analysis and is partially validated by clinical specimens from PAAD. Third, we found that the expression level of SNAI2 is associated with tumor immunity, but the specific mechanism of action is still unclear and needs to be further explored. Nonetheless, our pan-cancer research gives a thorough understanding of SNAI2’s tumor-targeting mechanism.

## Conclusion

In summary, our study indicated that SNAI2 expression varies in different tumors and cells. And high-SNAI2 expression was associated with poor survival outcomes and disease progression. In addition, SNAI2 levels were also found to be closely related to immune infiltrating cell expression, immune checkpoint gene expression, TMB, MSI, MMR gene, DNA methylation, etc. Our careful analysis of this study provided insights into the significant immunological advantages of SNAI2 as a pan-cancer prognostic and immunotherapy biomarker. This not only provided powerful new insights into the development of future immunotherapy and diagnostic studies evidence but also provides new treatments for cancer patients.

## Data availability statement

Publicly available datasets were analyzed in this study. This data can be found here: The public databases include the TCGA database (https://gdc.cancer.gov/); GTEx database ((https://gtexport.org/home/); THPA database (https://www.proteinatlas.org/); UCSC Xena database (https://xenabrowser.net/datapages/); Networks Functional Enrichment Analysis (https://string-db.org/); and cBioPortal web (https://www.cbioportal.org/).

## Ethics statement

The studies involving human participants were reviewed and approved by Ethics Committee of The Second Affiliated Hospital of Nanchang University. The patients/participants provided their written informed consent to participate in this study.

## Author contributions

DZ and YS contributed to the conception and design of the study. DZ wrote the first draft of the manuscript. ZJ, YZ, and JH performed the statistical and bioinformatic analysis. XS conducted figure visualization of the manuscript. All authors contributed to the article and approved the submitted version.

## Supplementary material

Supplementary Figure 1 Correlation analysis between expression levels of SNAI2 and ImmuneScore in pan-cancer.

Supplementary Figure 2 Correlation analysis between expression levels of SNAI2 and EstimateScore in pan-cancer.

Supplementary Figure 3 Correlation analysis between expression levels of SNAI2 and StromalScore in pan-cancer.

Supplementary Figure 4 Correlation analysis between SNAI2 expression in pan-cancer and the number of tumors neoantigens in pan-cancer.

Supplementary Figure 5 The correlation analysis of SNAI2 expression with MMR genes and DNA methyltransferases. **(A)** The Spearman’s correlation analysis of SNAI2 expression with MMR genes in cancers. **(B)** The Spearman’s correlation analysis of SNAI2 expression with DNA methyltransferases in various cancers. **p* < 0.05, ***p* < 0.01, ****p* < 0.001, ns, no significance.

Supplementary Figure 6 Overexpression of SNAI2 promotes cell proliferation and promotes cell apoptosis of pancreatic cancer cells. **(A)** The protein and mRNA levels of SNAI2 in pancreatic cancer cells (PANC-1, SW1990, BxPC-1, SW-979) and the H6C7 line. **(B)** Western blotting and qRT-PCR analyses of SNAI2 expression levels in SW1990 cells transfected with vector or His-SNAI2 plasmid. **(C)** CCK-8 assay showing the proliferation ability of SW1990 cells following overexpression of SNAI2. **(D)** Representative images (left) and quantification (right) of colony formation assays of SW1990 cells transfected with vector or His-SNAI2 plasmids. **(E)** Representative images (left) and quantification (right) of EDU assays of SW1990 cells transfected with vector or His-SNAI2 plasmids. Scale bar, 50 μm. **(F)** Representative images of the transwell invasion assays of SW1990 cells transfected with vector or His-SNAI2 plasmids. Scale bar, 50 μm. **(G)**. Western blotting showed the changes of EMT proteins in SW1990 cells transfected with vector or His-SNAI2 plasmids. All data are presented as the mean ± SD of three independent experiments. **p* < 0.05, ***p* < 0.01, ns, no significance.
