# Supplementary figures and images for: Comprehensively prognostic and immunological analysis of snail family transcriptional repressor 2 in pan-cancer and identification in pancreatic carcinoma
Source: Front Immunol. 2023 May 11;14:1117585. doi: 10.3389/fimmu.2023.1117585 (PMC10213725; doi:10.3389/fimmu.2023.1117585)

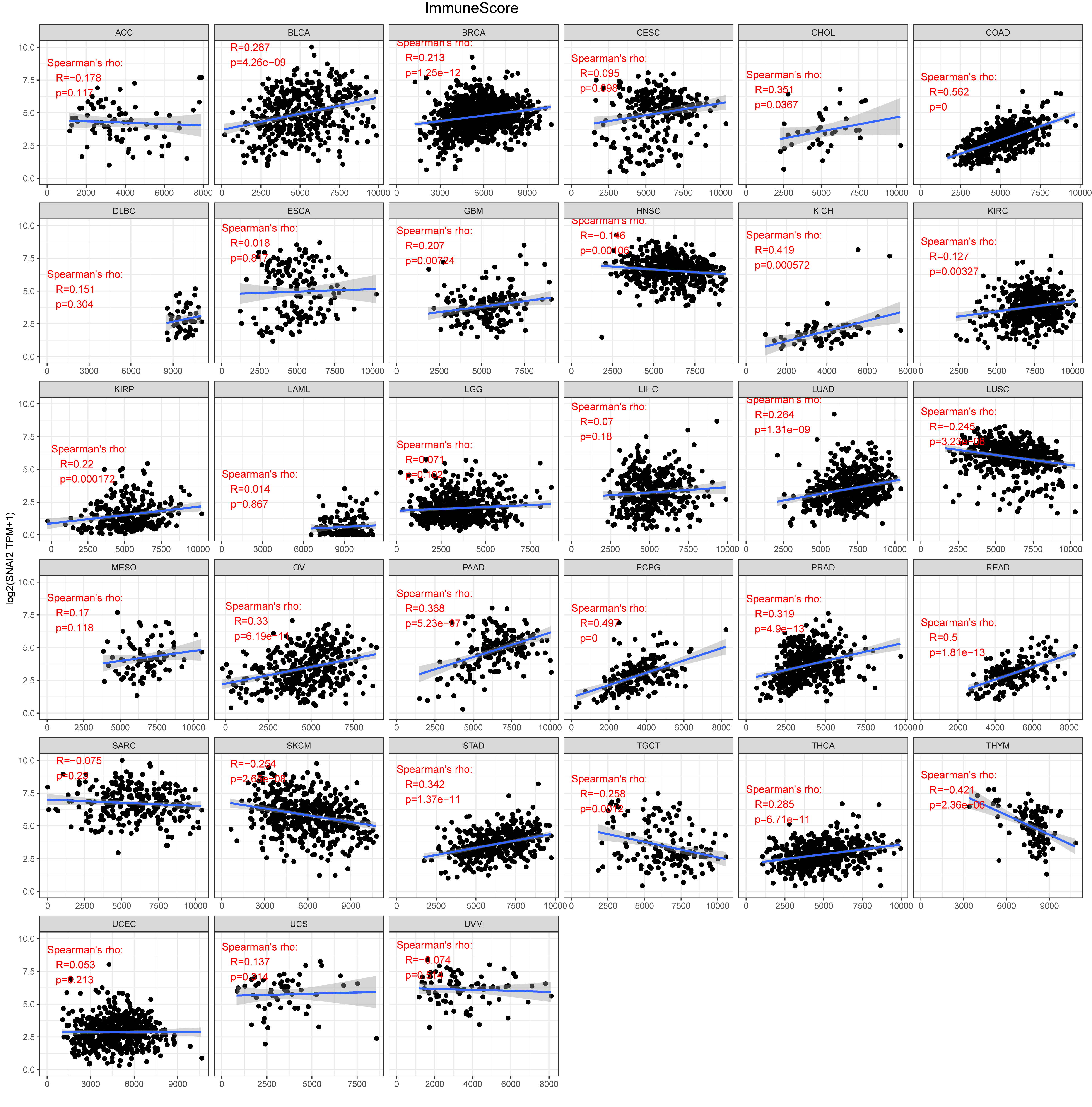

Supplement: Supplementary Figure 1 — Correlation analysis between expression levels of SNAI2 and ImmuneScore in pan-cancer. [file Image_1.tif]

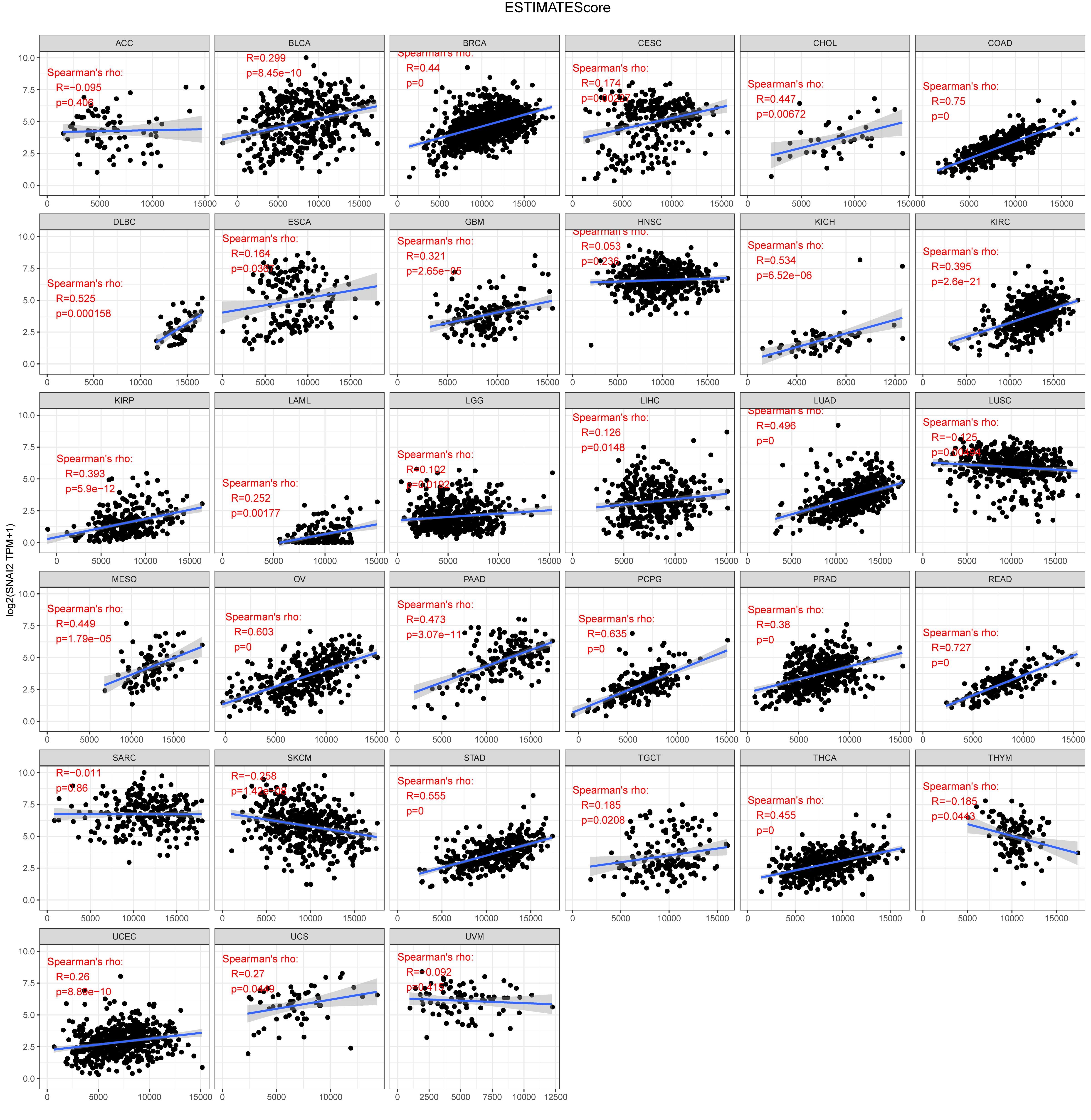

Supplement: Supplementary Figure 2 — Correlation analysis between expression levels of SNAI2 and EstimateScore in pan-cancer. [file Image_2.tif]

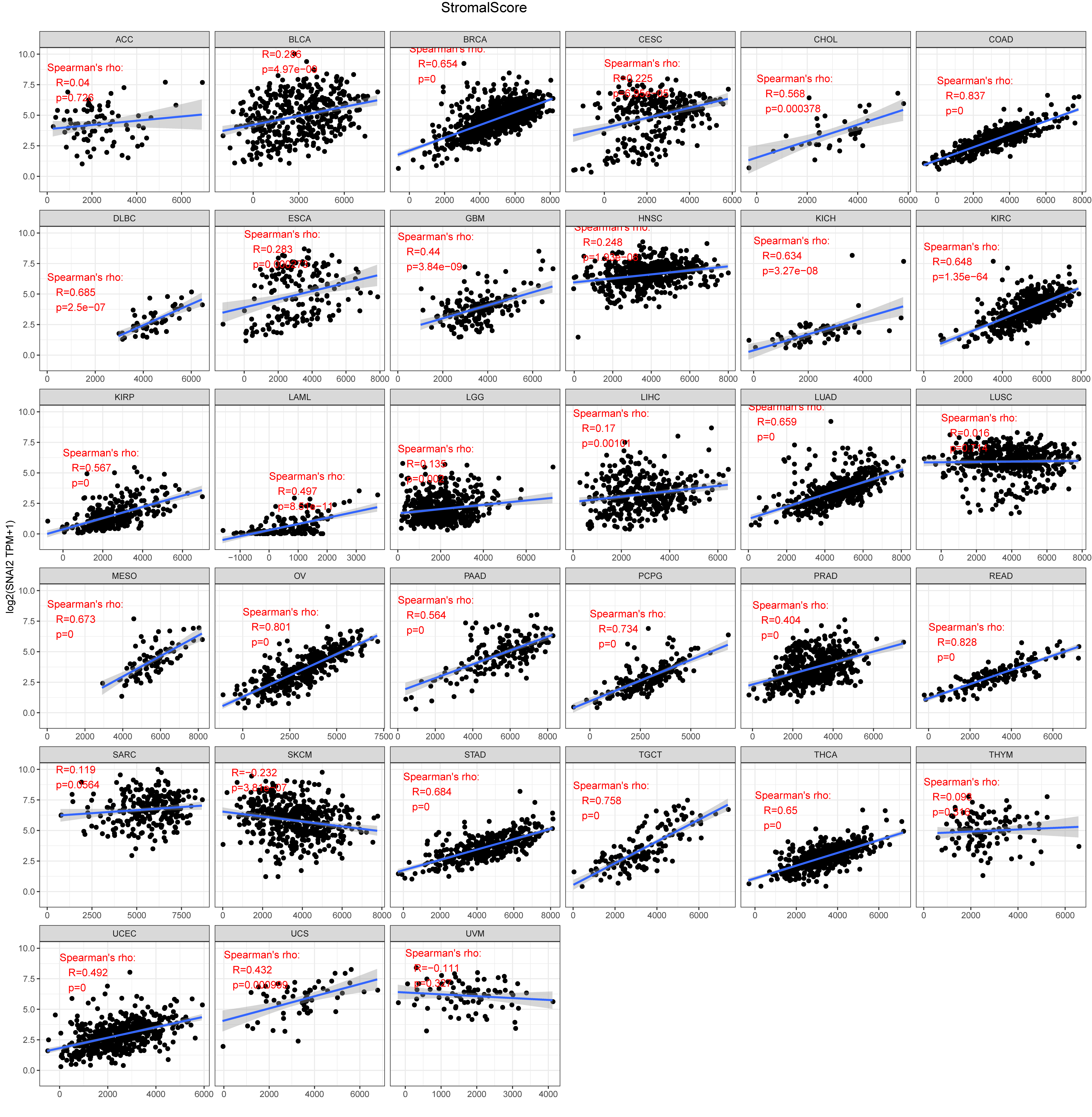

Supplement: Supplementary Figure 3 — Correlation analysis between expression levels of SNAI2 and StromalScore in pan-cancer. [file Image_3.tif]

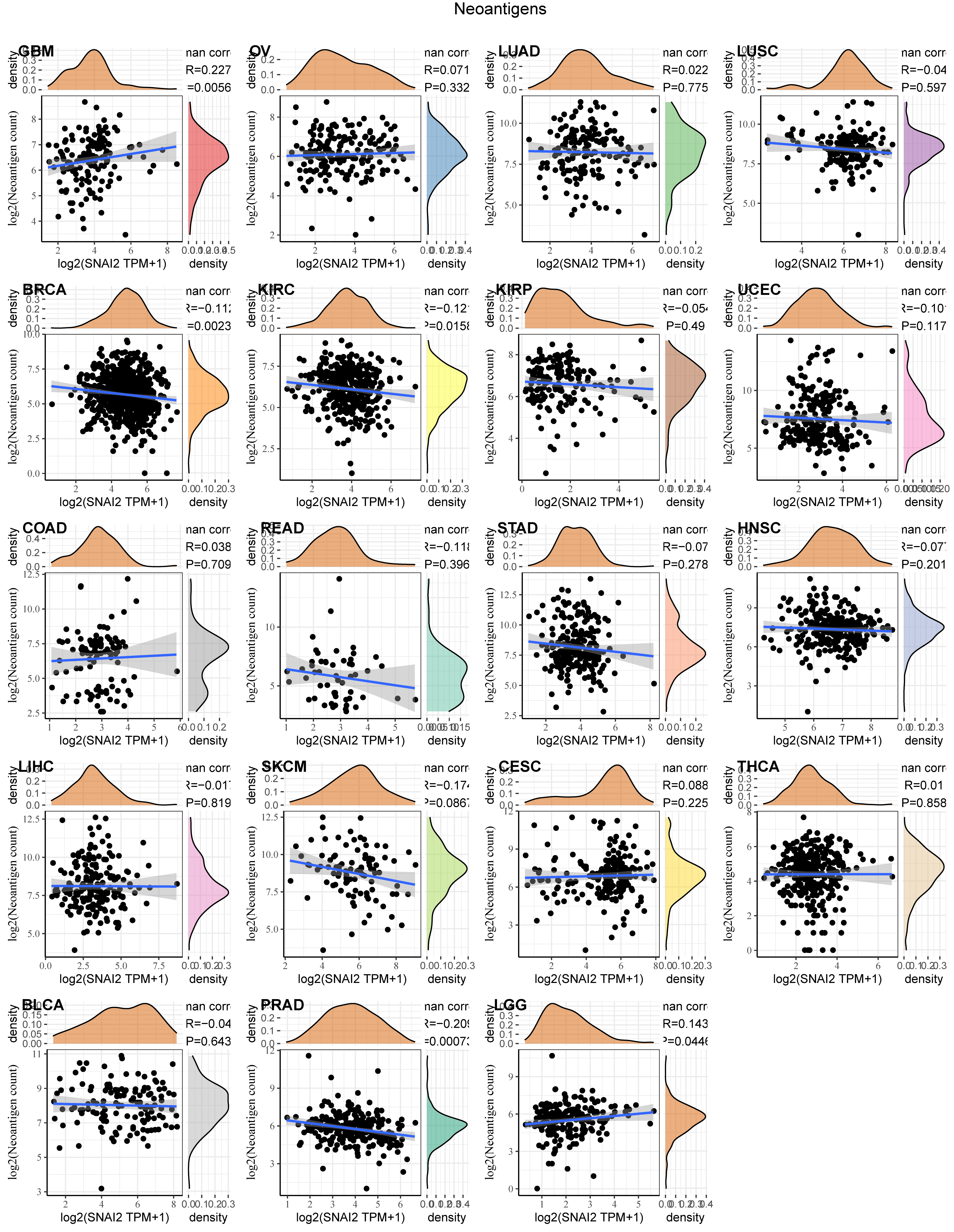

Supplement: Supplementary Figure 4 — Correlation analysis between SNAI2 expression in pan-cancer and the number of tumors neoantigens in pan-cancer. [file Image_4.tif]

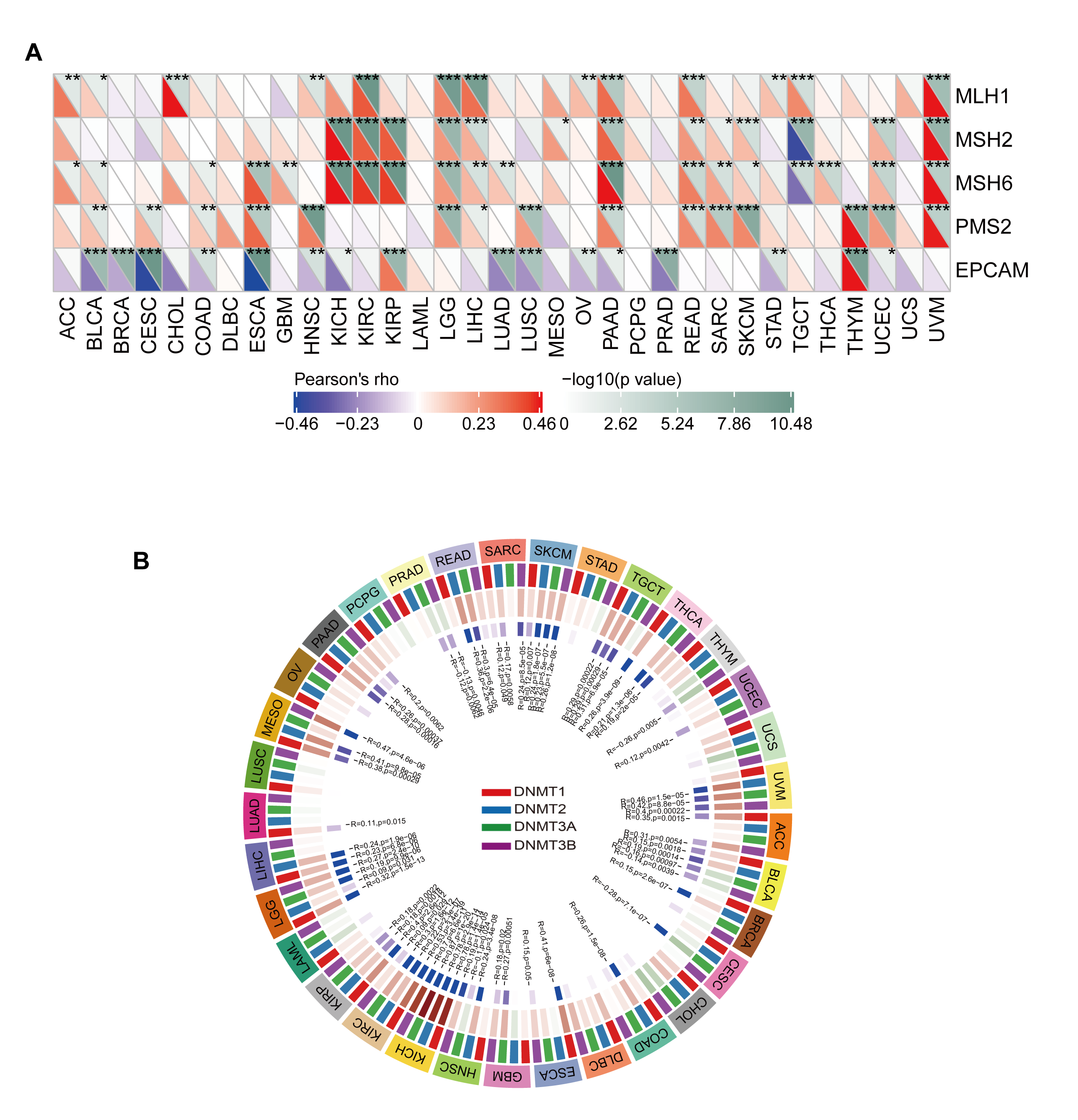

Supplement: Supplementary Figure 5 — The correlation analysis of SNAI2 expression with MMR genes and DNA methyltransferases. (A) The Spearman’s correlation analysis of SNAI2 expression with MMR genes in cancers. (B) The Spearman’s correlation analysis of SNAI2 expression with DNA methyltransferases in various cancers. *p < 0.05, **p < 0.01, ***p < 0.001, ns, no significance. [file Image_5.tif]

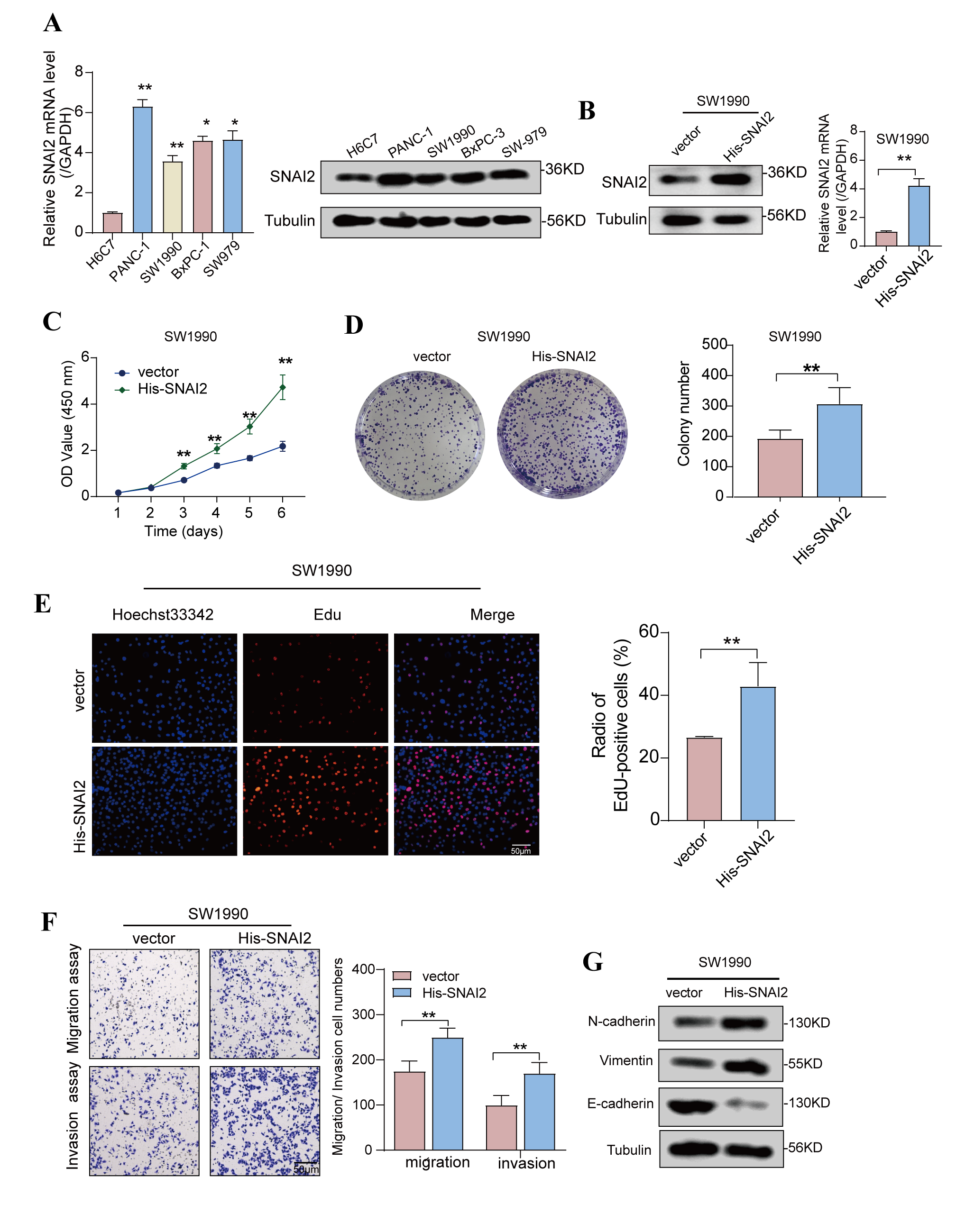

Supplement: Supplementary Figure 6 — Overexpression of SNAI2 promotes cell proliferation and promotes cell apoptosis of pancreatic cancer cells. (A) The protein and mRNA levels of SNAI2 in pancreatic cancer cells (PANC-1, SW1990, BxPC-1, SW-979) and the H6C7 line. (B) Western blotting and qRT-PCR analyses of SNAI2 expression levels in SW1990 cells transfected with vector or His-SNAI2 plasmid. (C) CCK-8 assay showing the proliferation ability of SW1990 cells following overexpression of SNAI2. (D) Representative images (left) and quantification (right) of colony formation assays of SW1990 cells transfected with vector or His-SNAI2 plasmids. (E) Representative images (left) and quantification (right) of EDU assays of SW1990 cells transfected with vector or His-SNAI2 plasmids. Scale bar, 50 μm. (F) Representative images of the transwell invasion assays of SW1990 cells transfected with vector or His-SNAI2 plasmids. Scale bar, 50 μm. (G). Western blotting showed the changes of EMT proteins in SW1990 cells transfected with vector or His-SNAI2 plasmids. All data are presented as the mean ± SD of three independent experiments. *p < 0.05, **p < 0.01, ns, no significance. [file Image_6.tif]
